# Modulation of Apoptosis Pathways by Oxidative Stress and Autophagy in *β* Cells

**DOI:** 10.1155/2012/647914

**Published:** 2012-03-12

**Authors:** Maorong Wang, Mia Crager, Subbiah Pugazhenthi

**Affiliations:** ^1^Section of Endocrinology, Veterans Affairs Medical Center, Denver, CO 80220, USA; ^2^Department of Medicine, University of Colorado Denver School of Medicine, Aurora, CO 80045, USA

## Abstract

Human islets isolated for transplantation are exposed to multiple stresses including oxidative stress and hypoxia resulting in significant loss of functional *β* cell mass. In this study we examined the modulation of apoptosis pathway genes in islets exposed to hydrogen peroxide, peroxynitrite, hypoxia, and cytokines. We observed parallel induction of pro- and antiapoptotic pathways and identified several novel genes including BFAR, CARD8, BNIP3, and CIDE-A. As BNIP3 is an inducer of autophagy, we examined this pathway in MIN6 cells, a mouse beta cell line and in human islets. Culture of MIN6 cells under low serum conditions increased the levels of several proteins in autophagy pathway, including ATG4, Beclin 1, LAMP-2, and UVRAG. Amino acid deprivation led to induction of autophagy in human islets. Preconditioning of islets with inducers of autophagy protected them from hypoxia-induced apoptosis. However, induction of autophagy during hypoxia exacerbated apoptotic cell death. ER stress led to induction of autophagy and apoptosis in *β* cells. Overexpression of MnSOD, an enzyme that scavenges free radicals, resulted in protection of MIN6 cells from cytokine-induced apoptosis. Ceramide, a mediator of cytokine-induced injury, reduced the active phosphorylated form of Akt and downregulated the promoter activity of the antiapoptotic gene bcl-2. Furthermore, cytokine-stimulated JNK pathway downregulated the bcl-2 promoter activity which was reversed by preincubation with SP600125, a JNK inhibitor. Our findings suggest that *β* cell apoptosis by multiple stresses in islets isolated for transplantation is the result of orchestrated gene expression in apoptosis pathway.

## 1. Introduction

The major pathways of apoptosis are the extrinsic pathway, initiated by Fas and other death receptors resulting in the activation of caspase-8, and the intrinsic mitochondrial pathway, regulated by Bcl-2 family of proteins leading to the activation of caspase-9 [1, 2]. These two pathways converge with the activation of caspase-3. Both apoptotic pathways are involved in *β* cell death in type 1 and type 2 diabetes [3]. Fas (CD95/APO-1) is a 36-kD death receptor protein that initiates apoptosis in many cell types when cross-linked to Fas ligand (FasL/CD95L) [4]. A histological study of human diabetic pancreas biopsies has demonstrated Fas expression on *β* cells and FasL expression on the infiltrating cells [5]. However, the intrinsic mitochondrial pathway, regulated by the Bcl-2 family of proteins, consisting of proapoptotic (Bax and Bak1) and antiapoptotic (Bcl-2, Bcl-xL, Mcl-1, etc.) proteins [6], has been shown to play a predominant role in the loss of isolated islets [7]. Imbalance between these two groups of proteins results in the release of cytochrome c, which activates caspase-9 [2]. BH3-only proteins, a subset of proapoptotic proteins, act as sensors of cellular stress [8, 9]. Members of this family include Bad, Bid, Bmf, Hrk, Bim, Bik, Noxa, and Puma. They induce apoptosis by activating proapoptotic proteins or by neutralizing antiapoptotic proteins. Bid cleaved by caspase-8 translocates to mitochondria and causes cytochrome c release, thus linking the two pathways of apoptosis [10].

Autophagy is a lysosomal degradative pathway that provides energy through self-digestion under conditions of starvation. During oxidative stress, autophagy serves as a defense mechanism to clear oxidatively damaged proteins and organelles [11]. There are three major pathways of autophagy: (1) chaperone-mediated autophagy (CMA), which is found in mammalian cells alone and degrades cytosolic proteins selectively; (2) microautophagy, whereby lysosomes directly engulf cytosolic constituents through invaginations of the lysosomal membrane; and (3) macroautophagy (referred to as autophagy), in which cytosolic contents including organelles and proteins are sequestered within double-membrane structures called autophagosomes that fuse with lysosomes and lead to degradation. A series of maturation steps involving the ATG family of proteins are involved in the formation of autophagosomes. Among the 30 ATG genes identified in yeast, 11 (ATG1, 3–10, 12, and 16) have orthologs in mammalian cells. Autophagic vesicles contain multiple proteins, including Type (or Class) III PI3 kinase (vps34), Beclin 1 (ATG6), UVRAG, and Ambra. An important step in the formation of autophagosomes is the conjugation of LC3 (ATG8) with phosphatidylethoanlamine to form LC3-PE (LC3-II) which is a standard marker for autophagy. Extensive autophagy could lead to type 2 cell death, a second mode of programmed cell death [12]. Aged insulin-secreting granules in *β* cells are degraded by crinophagy [13]. An imbalance between insulin production and secretion, which is likely to occur in type 2 diabetes, induces autophagy to degrade accumulated insulin granules [14]. Ubiquitinated protein aggregates that accumulate in *β* cells of islets in obese Zucker rats have been shown to stimulate autophagy [15]. Increased autophagic activity has been observed in the islets of Rab3A^−/−^ mice which display a defect in insulin secretion [13].

Islets are clusters of different cell types including *α*, *β*, *δ*, and PP cells, with insulin-producing *β* cells being the major component (70–80%). The blood vessels inside islets are essential for the supply of oxygen, nutrients, and secretion of hormones. These vessels are disrupted during the islet isolation process. Thus, islets are vulnerable to injury in the early stages after transplantation due to the delay in revascularization [16]. Even after revascularization, the vascular density is considerably less compared to endogenous islets [17]. The expression of genes associated with angiogenesis is decreased in diabetic transplant recipients, further delaying the revascularization process [18]. The molecular mechanism of apoptosis in *β* cells of islets in the transplantation setting is not clearly understood. The objective of the present study was to profile the expression of apoptosis pathway genes in human islets exposed to stresses associated with islet isolation and transplantation and to determine the role of stress-signaling pathways.

## 2. Experimental Procedures

### 2.1. Culture of Human Islets and MIN6 Cells

Human islets isolated from cadaveric donors were provided by Integrated Islet Distribution Program (IIDP). Islets with purity of 70% to 95% and the viability of 70%–95% were used. Islets were cultured in CMRL 1066 medium (Mediatech Inc., Hendon, VA) supplemented with human serum albumin (0.5%; Octapharma, Vienna, Austria) and nicotinamide (10 mM) and referred to as Miami medium. To induce hypoxia in islets, culture dishes were placed inside a modular incubation chamber (Billups Rothenberg, Del Mar, CA) and flushed with a gas supply of 1% oxygen, 5% CO_2_, and 94% N_2_ for 20 min. The chamber was then placed inside the cell culture incubator. MIN6 cells, a mouse pancreatic *β* cell line [19], obtained from Dr. Miyazaki (Kyoto University, Japan) were cultured in RPMI medium containing 10% FBS (Gemini Bioproducts, Sacramento, CA), 100 *μ*g/mL streptomycin (Gemini Bioproducts), 100 U/mL penicillin (Gemini Bioproducts), and 50 *μ*M *β*-mercaptoethanol (BME; Sigma Aldrich, St. Louis, MO) at 37°C in a humidified atmosphere of 5% CO_2_. Autophagy was induced in MIN6 cells by culturing them in low serum (0.1%) serum-containing medium and in human islets by amino acid deprivation.

### 2.2. Gene Expression Profiling of Apoptosis Pathways

Human islets isolated from donor pancreas were exposed to Hydrogen peroxide, peroxynitrite, hypoxia, or a combination of cytokines (IL-1*β*, TNF-*α*, and IFN-*γ*). A pathway-specific, PCR-based array (SABioscience, Frederick, MD) was performed to determine the expression of a panel of genes in apoptosis pathway (listed in Table 1). RNA samples following DNase treatment were converted to cDNA by the following procedure: 1 *μ*g of RNA was reverse transcribed in a 50 *μ*L reaction mixture with 125 U of Moloney Murine leukemia virus reverse transcriptase, 20 U RNasin ribonuclease inhibitor, 4 mM deoxyribonucleoside triphosphates, 5 mM MgCl_2_, 1X PCR Buffer II, and 0.5 *μ*g of random hexanucleotide primers (Applied Biosystems, Carlsbad, CA). The reaction mixture was sequentially incubated at 65°C for 5 min, 42°C for 60 min, and the reverse transcription reaction was stopped by heating to 95°C for 5 min and cooled to 4°C. The experimental cocktail was prepared by adding 102 *μ*L of the diluted cDNA to 1278 *μ*L of the RT^2^ qPCR master mix containing SYBR Green (SA Biosciences, Frederick, MD) and 1173 *μ*L H_2_O. 25 *μ*L of this cocktail was added to each well of the 96-well PCR array plate containing primers for the 84 genes in apoptotic pathway, five housekeeping control genes, and three RNA and PCR quality controls. Real-time PCR was performed in an ABI Prism 7700 sequence detector (Applied Biosystems, Foster City, CA). The thermal cycling conditions were 1 cycle of 10 minutes at 95°C followed by 40 cycles of 15 seconds at 95°C and of 1 minute at 60°C. After amplification, real-time data acquisition and analysis were performed through the Data Analysis Web Portal (SA Biosciences). Data analysis is based on the delta-delta Ct method with normalization of the raw data to GAPDH as described in the manufacturer's manual.

### 2.3. RNA Isolation and Real-Time Quantitative RT-PCR

Total RNA was isolated from treated islets. After DNAse treatment, RNA integrity was determined by capillary electrophoresis using RNA 6000 Nano LabChip. The mRNA levels of selected genes (BFAR, CARD8, CIDE-A, and BNIP3) identified in the expression profiling described previously were examined by real-time quantitative RT-PCR using Taqman probes. Assay on Demand (Applied Biosystems) was used for BNIP3. The sequence for primers and probes for other targets is as follows:

*BFAR*
Forward Primer: GGAGGACATCGTCACCAAGC
Reverse Primer: AGTATTTGACCAGGAACTCTCTCCA
TaqMan Probe: 5′-6FAM-TCTGGATCTTAAGGAGCCTACGTGGAAGCA-TAMRA-3′
*CARD8*
Forward Primer: GACTTCCGGTCGCCATGAT
Reverse Primer: ACCATTGAAGATGGCCCAGA
TaqMan Probe: 5′-6FAM-TGGGCGGTAAACGCGGTTAGTGC-TAMRA-3′

*CIDE-A*
Forward Primer: TCTTTCAGACCTTGGGAGACAAC
Reverse Primer: TGGCTGCCCGGCATC
TaqMan Probe: 5′-6FAM-CGCATTTCATGATCTTGGAAAAAGGACAGA-TAMRA-3′.

The PCR reactions were monitored in real time in an ABI Prism 7700 sequence detector (Perkin Elmer Corp./Applied Biosystems). After amplification, real-time data acquisition and analysis were performed.

### 2.4. Western Blot Analysis

MIN6 cells and human islets exposed to stress conditions were washed with ice-cold PBS and lysed with mammalian protein extraction reagent (M-PER, Pierce, Rockford, Illinois, USA) supplemented with phosphatase inhibitors (20 mM of sodium fluoride, 1 mM of sodium orthovanadate and 500 nM of okadaic acid) and protease inhibitors (Sigma P8340). Lysates were centrifuged at 20,800 ×g, and the protein concentration was determined in the supernatant samples by a dye-binding method [20]. Diluted samples containing equal amounts of protein were mixed with 2 X Laemmli sample buffer. The proteins were resolved on a 12% SDS-polyacrylamide gels. Following transfer to PVDF membranes (Millipore, Bedford, MA), the blots were blocked with TBST (20 mM Tris-HCl (pH 7.9), 8.5% NaCl, and 0.1% Tween 20) containing 5% non-fat dry milk at room temperature (RT) for 1 h and exposed to primary antibodies (1 : 1000; Cell Signaling, Danvers, MA) in TBST containing 5.0% BSA at 4°C overnight. The blots were washed with TBST and anti-rabbit IgG conjugated to alkaline phosphatase (Cell Signaling) was added for one hour at RT. After incubation in the presence of alkaline buffer (10 mM Tris-HCl (pH 9.5), 10 mM, NaCl and 1 mM MgCl_2_), signals were developed with CDP-Star reagent (New England Biolabs, Beverly, MA) and exposed to X-ray film. The intensity of bands was measured using Fluor-S MultiImager and Quantity One software from Bio-Rad.

### 2.5. Transfection Procedure

The activity of bcl-2 promoter (truncated with CRE site, −1640–1287) linked to firefly luciferase reporter gene (provided by Linda Boxer, Stanford University School of Medicine) was measured in cultured MIN6 cells by a transient transfection assay using the procedure described earlier [21]. Plasmids (2 *μ*g) and LipofectAMINE reagent 2000 (Invitrogen-Life Technologies, Carlsbad, CA) (4 *μ*L) were diluted separately in 100 *μ*L of Opti-MEM I, mixed, and incubated at room temperature for 20 min. A constitutively active renilla luciferase (pRL-TK-luc) was included to correct for transfection efficiency and for nonspecific actions of treatments on luciferase activity. The plasmid mixture were added to MIN6 cells cultured in 12 well dishes to about 70% confluence. The transfected MIN6 cells were cultured in low serum (0.1%) medium with appropriate treatment. The treated cells were washed with cold PBS and lysed with 100 *μ*L of reporter lysis buffer. The lysate was centrifuged (10,600 ×g; 20 min) to collect the supernatant. The activities of firefly luciferase and renilla luciferase were measured using a dual luciferase assay kit (Promega, Madison, WI). The ratios of these two luciferase activities were taken as the measure of bcl-2 promoter induction.

### 2.6. Immunocytochemistry

MIN6 cells were cultured in a Lab-Tek II Chamber Slide system; following transfection and treatment, they were fixed in 4% paraformaldehyde for 30 minutes at RT. After washing with PBS, fixed cells were permeabilized in PBS containing 0.2% Triton X-100 and 5% BSA for 90 minutes. The cells were incubated in the presence of antibodies for active caspase-9 or active caspase-3 (1 : 250) in 3% BSA at 4°C overnight, washed in PBS, and exposed to secondary antibodies linked to Cy3 (Jackson ImmunoResearch Laboratories, West Grove, Pennsylvania, USA) in 3% BSA for 90 minutes at RT. Cells were then washed in PBS, sealed with mounting medium and examined by digital deconvolution microscopy using a Zeiss Axioplan 2 microscope fitted with Cooke SensiCam^QE^ high performance CCD camera. Immunocytochemistry with islets was carried out by a similar procedure but with islets in suspension. Primary antibodies were at the dilution of 1 : 2000 for insulin and 1 : 500 for LC3-II and phosphorylated Akt. Appropriate secondary antibodies linked to Cy3 or FITC in 3% BSA were used. Washing steps were performed by centrifugation at 500 RPM for 5 min. After the final step of washing the fluorescent labeled islets in PBS, they were suspended in mounting medium and placed inside secure seal hybridization chambers for fluorescent microscopy. Images were taken in multiple *z*-planes and assembled together by digital deconvolution microscopy.

Statistical analysis was performed by one-way analysis of variance (ANOVA) with Dunnett's multiple comparison test. 

## 3. Results

### 3.1. Gene Expression Profiling of Apoptosis Pathways in Human Islets under Stress

We performed an apoptosis pathway-specific gene expression array with RNA isolated from human islets exposed to oxidative stress, hypoxia and proinflammatory cytokines. Genes in intrinsic and extrinsic pathways were induced in stressed islets (Table 2). For example, Fas, fas ligand, and TRAIL, important regulators of the extrinsic pathway, were induced (150–360%) by oxidative stress and cytokines. Bid that links both pathways was induced by 150–270%. The expression of CARD8, an inhibitor of caspase-9, a marker for the intrinsic pathway of apoptosis, was downregulated ~50% by oxidants, hypoxia, and cytokines. Downregulation of BRAF (60–73%), a critical signaling kinase, suggests that these islets are likely to be less responsive to growth factor-mediated cell survival pathways. Caspase-3, a marker for apoptosis, is activated by proteolytic cleavage. In this study, we also observed increased expression of caspase-3 by all the stresses tested. CIDE-A which causes DNA fragmentation was induced (140–280%) by oxidative stress and hypoxia. Interestingly, activation of a parallel cytoprotective pathway in response to stress was evident by the induction of several genes in the antiapoptotic pathway including, Bcl2A1, c-IAP2 (BIRC3), A20, and c-flip (CFLAR). BNIP3, an inducer of autophagy was induced by 160% and 460% by peroxynitrite and hypoxia, respectively. Our findings from gene expression profiling points to the complex nature of cell death pathway in islets exposed to stresses associated with islet isolation and transplantation.

### 3.2. Modulation of Apoptosis Pathway Genes in Islets

**Figure 1:**
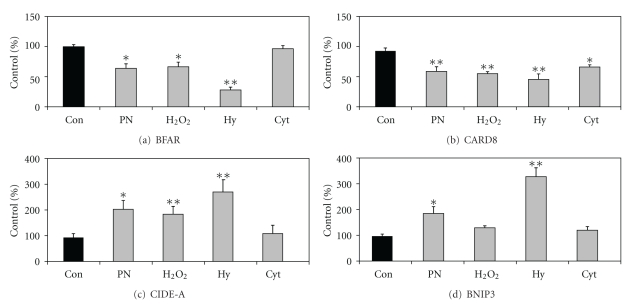
Stress-induced modulation of genes in apoptosis pathway. Human islets (2000 IEQ) cultured in Miami medium were exposed to multiple stresses including peroxynitrite (PN; 200 *μ*M), H_2_O_2_ (200 *μ*M), or a mixture of cytokines (Cyt; 2 ng/mL of IL-1*β*, 10 ng/mL of TNF-*α*, and 10 ng/mL of IFN-*γ*) for 24 h or cultured under hypoxic conditions (1% oxygen) for 8 h. RNA was isolated from treated islets for the RT-PCR analysis of BFAR, CARD8, CIDE-A, and BNIP3 using Taqman probes. Results are M ± SE of experiments with four independent batches of human islets. *P < 0.01; **P < 0.001 when compared to untreated control (Con).

Some of the genes including BFAR, CARD8, CIDE-A, and BNIP3 listed before have not been reported previously in the context of apoptosis in islets. Therefore, their induction was further confirmed by a more sensitive RT-PCR analysis using Taqman probes. BFAR** (**bifunctional apoptosis regulator) is a multidomain protein that interacts with members of the extrinsic and intrinsic apoptosis pathways and inhibits apoptosis. CARD8, a member of the caspase-associated recruitment domain (CARD) family, inhibits the activation of caspase-9. Levels of BFAR and CARD8 decreased by 35–40% when exposed to oxidative stress and by 50–70% and when incubated under hypoxic conditions (Figure 1). Cytokines decreased (30%) the expression of CARD8 but not BFAR. The mRNA levels of CIDE-A, a gene that acts downstream of caspases to mediate DNA fragmentation, increased by 95–180% in islets exposed to oxidative stress while cytokines did not have any effect on the expression of this gene. The expression of BNIP3 which plays an important role in mitochondrial autophagy increased in islets exposed to peroxynitrite (92%) and hypoxia (240%).

### 3.3. Induction of Autophagy in MIN6 Cells

**Figure 2:**
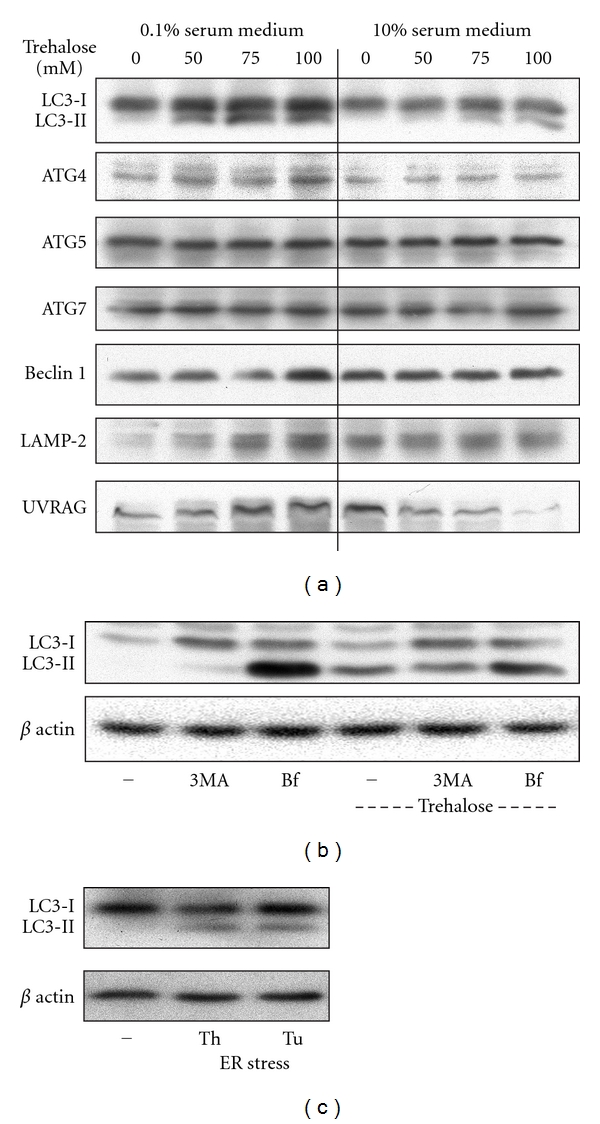
Induction of autophagy in MIN6 cells. (a) MIN6 cells grown to 60% confluence were incubated in low (0.1%) serum or regular (10%) serum medium in the presence of increasing concentrations of trehalose, an inducer of autophagy, for 24 h. (b) MIN6 cells were preincubated in the presence of 10 mM of 3-methyladenine (3MA) or 100 nM of bafilomycin A1 (Bf) for 20 min followed by exposure to 100 mM of trehalose for 24 h. (c) ER stress was induced in MIN6 cells by culturing them in the presence of thapsigargin (Th; 100 nM) or tunicamycin (Tu; 1 *μ*g/mL) for 24 h. Lysates of the treated cells ((a)–(c)) were analyzed by Western blotting for markers of autophagy. Representative images from three independent experiments are presented.

Autophagy is a self-digestive process by which cells generate energy during starvation and degrade damaged proteins and organelles. We examined autophagic pathways in MIN6 cells cultured under low serum conditions by Western blot analysis. A key step in the formation of autophagosomes is the conjugation of LC3 (LC3-I-18 kD) with phosphatidylethanolamine to form LC3-PE (LC3-II-16 kD), an important marker of autophagy. As shown in Figure 2(a), the levels of LC3-II increased with increasing concentrations of trehalose, particularly in low-serum medium. The levels of ATG4, LAMP-2, and UVRAG also increased in a similar manner. An increase in Beclin 1 was seen only at 100 mM of trehalose in low-serum medium. Exposure of *β* cells to the autophagy inhibitor 3-methyladenine (3MA) reduced the formation of LC3-II in the presence of trehalose (Figure 2(b)) whereas another inhibitor bafilomycin A1 (Bf) increased the accumulation of LC3-II both under basal conditions as well as following exposure to trehalose (Figure 2(b)) because it interferes at the late lysosomal degradation step leading to accumulation of autophagosomes. Furthermore, induction of ER stress with thapsigargin and tunicamycin (Calbiochem, La Jolla, CA) led to formation LC3-II (Figure 2(c)) suggesting a crosstalk between these two pathways.

### 3.4. Autophagic Preconditioning Protects Human Islets from Hypoxia-Induced Apoptosis

**Figure 3:**
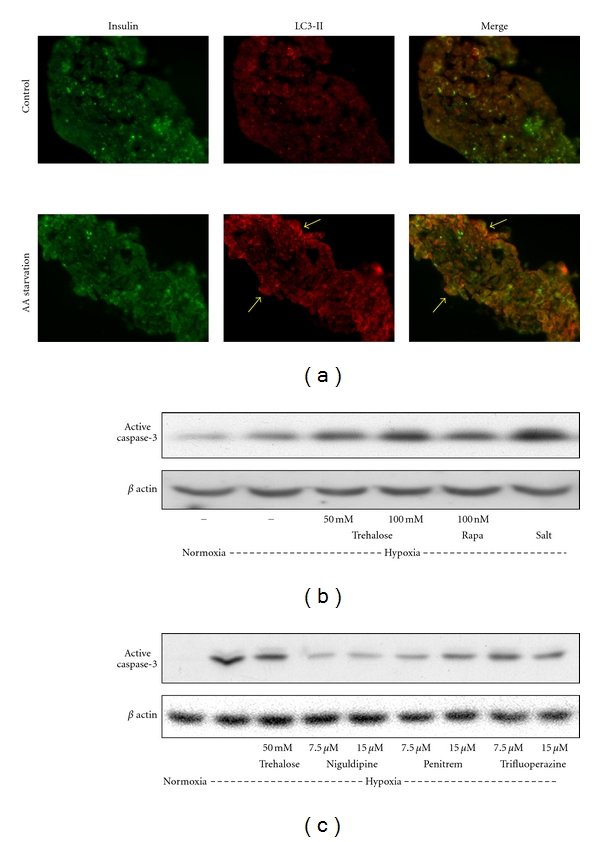
Induction of autophagy in human islets. (a) Human islets were incubated for 8 h in Miami medium (control) or in Hank's balanced salt solution for amino acid (AA) starvation. Treated islets were fixed in paraformaldehyde and embedded in OCT. Frozen sections (7 *μ*m thickness) were immunostained for insulin with FITC (Green) and for LC3-II with cy3 (red). Formation of autophagosomes was visualized by the punctated staining of LC3-II in *β* cells (arrows). (b) Human islets were cultured under normoxic or hypoxic conditions in the absence and presence of inducers of autophagy, trehalose, rapamycin (Rapa) or salt solution (Salt) for 8 h. Cell lysates were processed for Western blot analysis of the active form of caspase-3. Induction of autophagy during hypoxia exacerbated apoptosis in islets. (c) Human islets were preincubated with inducers of autophagy for 4 h. Following change of medium, the cells were cultured under normoxic or hypoxic conditions for 12 h, lysed and processed for the Western blot analysis of active caspase-3. Autophagic preconditioning protected islets from hypoxia-induced apoptosis. Representative images from experiments with three independent batches of human islets are presented.

Amino acid (AA) starvation of human islets by incubation in Hank's balanced salt solution induced autophagy in *β* cells as shown (arrows) by the punctated staining of LC3-II in insulin-positive cells (Figure 3(a)). Autophagy can be protective under conditions of starvation in transplanted islets till vascularization takes place. However, excessive autophagy is known to result in cell death through crosstalk with apoptosis. Therefore it becomes a dilemma from a therapeutic angle whether to inhibit or activate autophagy in transplanted islets. To test the strategies for modulation autophagy during hypoxia, we used trehalose, rapamycin, and amino acid starvation as inducers during hypoxia. When islets were exposed to hypoxia in the presence of autophagy inducers, there was exacerbation of apoptosis. Activation of caspase-3 during hypoxia increased further in the presence of trehalose (40–65%; *P* < 0.01), rapamycin (35%; *P* < 0.05), and salt solution (115%; *P* < 0.001) (Figure 3(b)) suggesting the need for moderate autophagy during hypoxia. Next we attempted autophagic preconditioning before exposure to hypoxia as an alternate strategy. A high-throughput screen of small molecules [22] has identified among inducers of autophagy, several FDF-approved drugs including Niguldipine, Penitrem A and trifluoperazine. Therefore we screened a number of compounds at different concentrations using MIN6 cells cultured under hypoxia conditions. Based on those results, we tested some of them in human islets by preincubation followed by culture under hypoxic conditions. Significant protection (*P* < 0.01) from hypoxia-induced apoptosis was seen with all the compounds tested (Figure 3(c)). Niguldipine showed that 75% decreases in active cleaved form of caspase-3 followed by penitrem (55%), trifluoperazine (40%), and trehalose (35%).

### 3.5. Antiapoptotic Actions of MnSOD and Exendin-4 in MIN6 Cells

**Figure 4:**
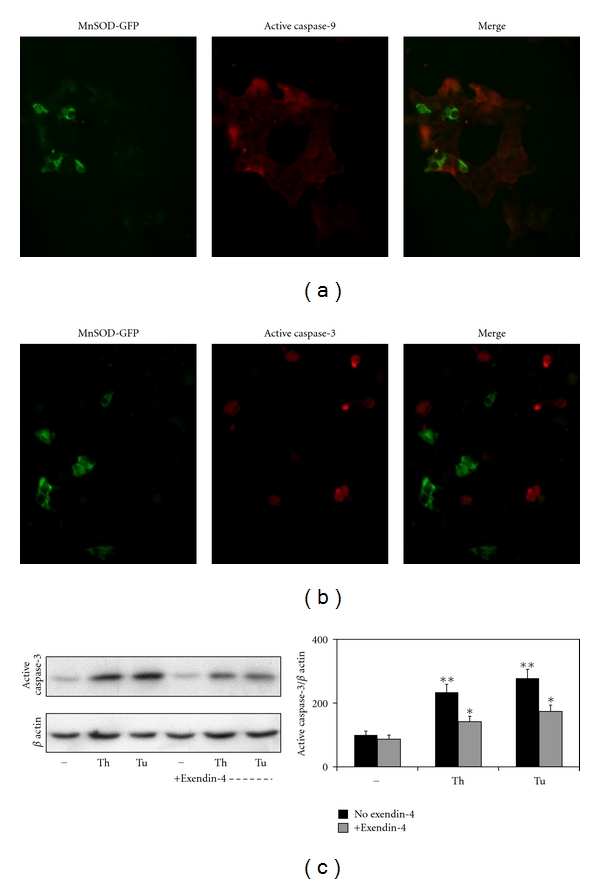
Antiapoptotic actions of MnSOD and exendin-4 in MIN6 cells: MIN6 cells cultured on chamber slides were transfected with cDNA encoding MnSOD-GFP chimeric protein. The transfected cells were exposed to a mixture of cytokines (2 ng of IL-1*β*, 10 ng of TNF-*α* and 10 ng of IFN-*γ*) for 24 h. The cells were fixed, permeabilized and probed with antibodies specific for the active cleaved fragment of caspase-9 (a) and caspase-3 (b). This was followed by probing with secondary antibodies linked to Cy3. The images were analyzed by digital deconvolution microscopy. The cells expressing MnSOD (green) are protected from cytokine-induced apoptosis. Images from three independent experiments are presented. (c) MIN6 cells were cultured in the absence and presence of exendin-4 (50 nM). ER stress was induced by exposing the cells to thapsigargin (Th; 100 nM) or tunicamycin (Tu; 1 *μ*g/mL) for 24 h. Lysates of the treated cells were analyzed by Western blotting for active caspase-3 and *β* actin. Band intensities were quantitated by scanning. Results are M ± SE of three independent experiments. **P < 0.001 when compared to untreated control. *P < 0.01 versus respective ER stress control in the absence of exendin-4.

MnSOD is an antioxidant enzyme that plays an important role in scavenging free radicals generated by oxidative stress. To determine the role of oxidative stress in cytokine-mediated *β* cell apoptosis, a cDNA encoding MnSOD-GFP chimeric protein was overexpressed in MIN6 cells followed by exposure to a mixture of cytokines (IL-1*β*, TNF-*α*, and IFN-*γ*) for 48 h. Activation of caspase-9, a marker for the intrinsic pathway of apoptosis, and activation of caspase 3, a general marker for apoptosis, were examined in treated cells by immunofluorescent staining. Cytokines induced apoptosis by the intrinsic pathway in MIN6 cells as shown by the staining for the active forms of caspase-9 (Figure 4(a)) and caspase-3 (Figure 4(b)) with cy3 (red). MnSOD-expressing cells were identified by the green fluorescence. Staining for active caspase-9 and -3 did not colocalize with MnSOD-GFP-expressing cells suggesting that these cells are protected from cytokine-induced apoptosis. At least 1000 GFP-expressing cells were examined for each experiment. We also tested the cytoprotective action of exendin-4, a glucagon-like peptide- (GLP-1) analogue, in MIN6 cells following induction of ER stress. Exposure of MIN6 cells to thapsigargin and tunicamycin, inducers of ER stress, caused apoptosis as shown by 135–180% (*P* < 0.001) increases in the levels of active caspase-3 (Figure 4(c)). Preincubation of MIN6 cells with exendin-4 resulted in significant (*P* < 0.01) protection with 40% decrease in the activation of caspase-3.

### 3.6. Cytokine-Generated Ceramides Interfere with Activation of Akt and CREB

**Figure 5:**
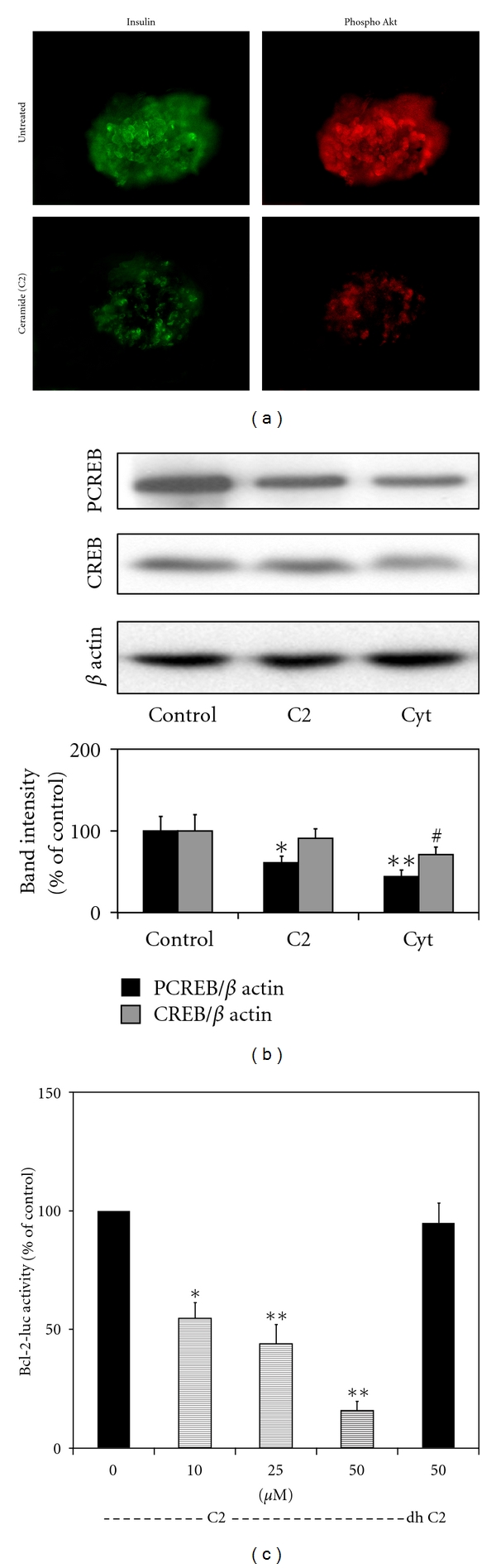
Ceramide is a mediator of cytokine-mediated CREB downregulation. (a) Human islets were exposed to C2 (10 *μ*M), a synthetic ceramide, for 24 h. The islets were fixed and immunostained for phospho Akt (Thr 308). The images were analyzed by digital deconvolution microscopy. Decrease in the active form of Akt was observed in ceramide-treated islets. (b) Islets were incubated in the absence and presence of C2 and a combination of cytokines for 24 h and processed for the Western blot analysis of phosphorylated form of CREB, total CREB and *β* actin. Band intensities were quantitated by scanning. The results are M ± SE of 3 independent experiments. *P < 0.01; **P < 0.001; ^#^ *P* < 0.05 compared to untreated control. Active form of CREB was decreased by both C2 and cytokines. (c) MIN6 cells were transfected with a CRE site-containing bcl-2 promoter linked to a firefly luciferase reporter and a constitutively active renilla luciferase (for transfection efficiency). The transfected cells were exposed to increasing concentrations of C2 and 50 *μ*M of an inactive analogue (dh) for 24 h. The cells were processed for luciferase activities using a dual luciferase assay kit. The ratio of firefly and renilla luciferase activities was taken as a measure of bcl-2 promoter activity. Bcl-2 promoter activity was inhibited by the ceramide. The results are M ± SE of 4 independent experiments. *P < 0.01; **P < 0.001 compared to untreated control.

Ceramides generated by cytokines are known to decrease Akt phosphorylation [23]. Akt plays a significant role in CREB phosphorylation and activation, leading to improved cell survival [24]. Therefore, human islets were exposed to 10 *μ*M of synthetic ceramide (C2) and the active phosphorylated form of Akt was immunofluorescently stained with Cy3 (red) along with staining for insulin with FITC (green). Treatment with C2 resulted in decrease in the levels of active form of Akt (Figure 5(a)). Western blot analysis of human islets treated with C2 or a combination of IL-1*β*, TNF-*α*, and IFN-*γ* showed 40–55% decrease in PCREB, the active phosphorylated form of CREB (Figure 5(b)). There was also a modest decrease (*P* < 0.05) in CREB levels in cytokine-treated islets. The activity of CRE-site containing bcl-2 promoter was also inhibited in a dose-dependent manner by C2 whereas the inactive analogue (dh C-2) did not affect the reporter activity (Figure 5(c)). We have previously reported similar downregulation of bcl-2 promoter by cytokines [25]. Thus a synthetic ceramide mimics the action of cytokines on *β* cell apoptosis.

### 3.7. Activation of JNK Pathway by Cytokines Plays a Role in Downregulation of Bcl-2 Promoter Activity

**Figure 6:**
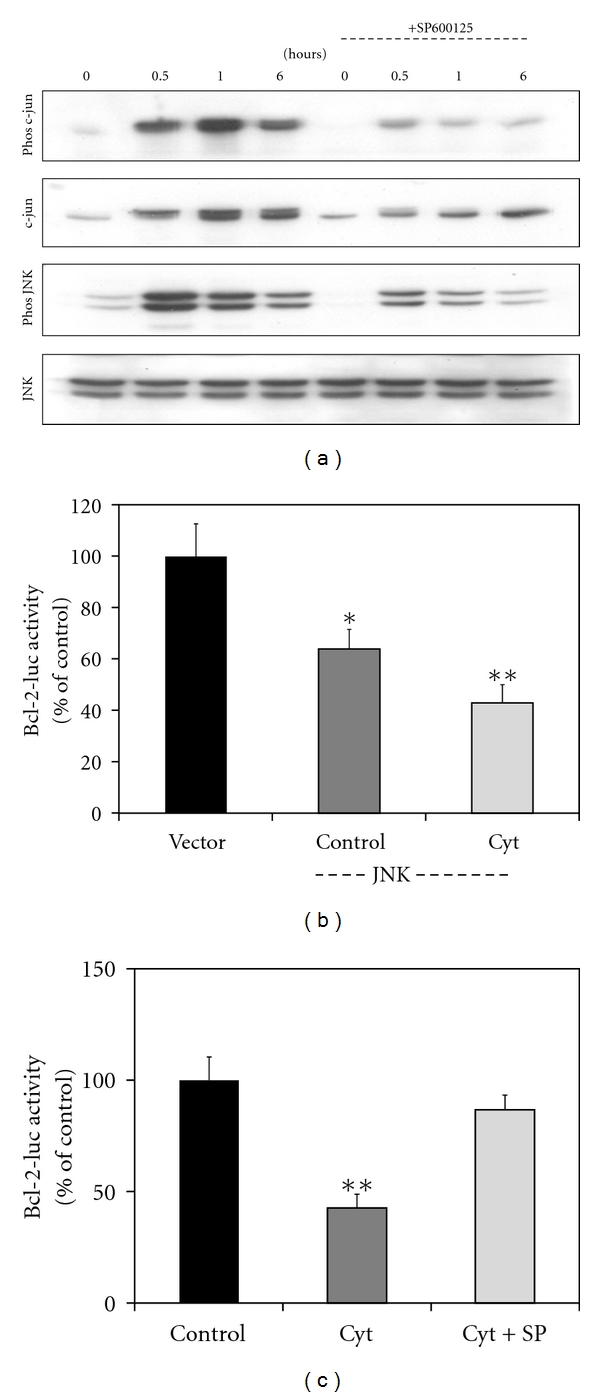
Activation of JNK by cytokines. (a) MIN6 cells were incubated in the absence and presence of 20 *μ*M JNK inhibitor, SP600125 and/or a combination of cytokines, IL-1*β* (2 ng/mL) TNF-*α* (10 ng/mL) and IFN-*γ* (10 ng/mL) for the indicated time periods. The treated cells were processed for the Western blot analysis of phosphorylated (Phos) c-jun, total c-jun, phosphorylated (Phos) JNK and total JNK. ((b) and (c)) MIN6 cells were transfected with a CRE site-containing bcl-2 promoter linked to a firefly luciferase reporter and a constitutively active renilla luciferase. A combination of plasmid mixture encoding the JNK isozymes or their vector was also included for one experiment (b). Transfected cells were incubated in the absence and presence of a combination of cytokines and JNK inhibitor (SP600125) as indicated for 24 h followed by the assay for luciferases. Bcl-2 promoter activity was inhibited by cytokines and JNK isozymes. Cytokine action on bcl-2 promoter activity was blocked by the JNK inhibitor. The results are M ± SE of 4 independent experiments. *P < 0.01; **P < 0.001 versus untreated control.

Cytokines strongly stimulate the signaling pathways leading to JNK activation. JNK-mediated activation of the transcription factor c-jun plays a critical role in inducing stress-associated genes in *β* cells [26]. A combination of cytokines strongly increased the active phosphorylated forms of JNK isozymes with the peak activation (*P* < 0.001) being observed at 30 min (Figure 6(a)). The levels of total JNK remained the same during the 6 h incubation period. Activation of JNK led to increases in the active phosphorylated form of c-jun. Maximum activation (370%) was seen at 1 h. The levels of c-jun also increased (110–235%) during 6 h exposure to cytokines as it is an autoregulated gene. Preincubation with the JNK inhibitor resulted in significant (*P* < 0.01) decreases in the phosphorylation of c-jun and in the levels of c-jun. As SP600125 also acts on kinases upstream of JNK, the phosphorylation of JNK itself was reduced. The bcl-2 promoter activity was inhibited when a combination of JNK isozymes was also expressed (Figure 6(b)). Cytokines further enhanced the downregulation of bcl-2 promoter activity by JNK isozymes whereas JNK inhibitor blocked cytokine action on bcl-2 promoter (Figure 6(c)).

## 4. Discussion

Oxidative stress is known to play an important role in *β* cell death in diabetes and in transplanted islets. Autophagy is a physiological mechanism that protects *β* cells under conditions of starvation and hypoxia. However, excessive autophagy resulting from oxidative stress can lead to *β* cell death by crosstalk with apoptosis pathway. In this study, we report the complex modulation of apoptosis pathway genes in human islets exposed to oxidative stress, hypoxia, and cytokines suggesting interactions between pathways involved in cell death as well as cell survival. We demonstrate that autophagic preconditioning of human islets leads to protection against hypoxia-induced apoptosis. Inducers of ER stress were also found to activate autophagy and apoptosis in *β* cells.

Pathway-specific, PCR-based arrays represent a novel technique to determine the expression of a family of genes associated with a specific pathway. We examined the expression of apoptosis pathway genes in human islets exposed to multiple stresses associated with diabetes, islet isolation, and transplantation. We observed significant increases in the expression of genes that are involved in opposing pathways of cell survival and cell death. It is also important to mention four genes, namely BFAR, CARD8, CIDE-A, and BNIP3, as their role in *β* cell apoptosis has not been reported previously. BFAR is a multidomain protein that improves cell survival by interacting with members of extrinsic and intrinsic pathways of apoptosis. Caspase-associated recruitment domain (CARD) family of proteins participates in the activation or inhibition of caspases. CARD8 inhibits the activation of caspase-9. The levels of both BFAR and CARD8 decreased significantly in islets exposed to oxidative stress and hypoxia, suggesting a decrease in antiapoptotic defense in islets. Levels of CIDE-A, a gene that acts downstream of caspases to mediate DNA fragmentation, increased 2- to 3-folds in islets exposed to stress, suggesting late stages of apoptosis. The expression of BNIP3 which plays an important role in autophagy by disrupting the interaction between Beclin 1 and Bcl-2 was induced by peroxynitrite and hypoxia. BNIP3 promotes mitochondrial autophagy as an adaptive response to hypoxia [27].

**Figure 7:**
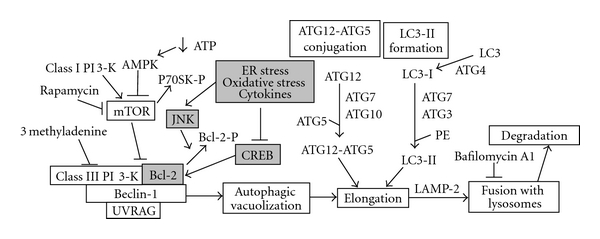
Interactions of signaling by ER stress, oxidative stress, and cytokines with the pathways of autophagy: Induction of autophagy requires the assembly of Beclin-1 with class III PI 3 kinase (Vps34) and UVRAG. Rapamycin is an activator of autophagy as it is negatively regulated by class I PI 3 kinase through mammalin target of rapamycin (mTOR). The elongation step involves two ubiquitin-like conjugation system. ATG12 is activated by E1-like ATG7, transferred to E2-like ATG10 and conjugated to ATG5. Similarly, LC3-I is activated by E1-like ATG7, transferred to E2-like ATG3, and conjugated to phosphatidylethanolamine (PE). Inhibitors of autophagy include 3 methyl adenine which inhibits class III PI 3-K and bafilomycin A1 which inhibits the formation of autophagolysosome. Interaction of Bcl-2 with Beclin-1 keeps the autophagic pathway under control. CREB induces the expression of Bcl-2 which is downregulated by ER stress, oxidative stress and cytokines through JNK.

Several studies have reported significant interactions between the pathways of apoptosis and autophagy [12, 28, 29]. Apoptosis and autophagy could act as partners to induce cell death. For example, death-associated protein kinase (DAPK) family members, activated by cytokines, are involved in cell death by apoptosis as well as autophagy [12]. Conjugation of ATG5 with ATG12 is an important step in the formation of autophagosomes during basal autophagy. However, when there is excessive autophagy, ATG5 has been reported to interact with components of pathways of apoptosis. Calpain cleaves ATG5 to generate a truncated form of this protein which translocates to mitochondria and induces the release of cytochrome c from mitochondria to activate the intrinsic pathway. The antiapoptotic proteins Bcl-2 and Bcl-xL bind and inhibit Beclin 1-mediated autophagy [30]. This interaction results in the inhibition of autophagy because Beclin 1 will not be able to bind to class III PI 3-kinase (VPS34) and UVRAG, an important step in the initiation of autophagosome formation. Essentially, Bcl-2 keeps autophagy under control (Figure 7). We have demonstrated previously that Bcl-2 levels are regulated by CREB [25]. Therefore, under conditions of CREB downregulation, the pathway of autophagy can be dysregulated. In addition, JNK1-mediated phosphorylation of Bcl-2 also results in the disruption of its interaction with Beclin 1, leading to activation of autophagy [31]. Thus physiological autophagy which improves cell survival can become uncontrolled when *β* cells are exposed to oxidative stress and cytokines.

Autophagy is beneficial to islets in the transplantation setting because it clears organelles damaged by oxidative stress generated during islet isolation and provides energy during starvation (hypoxia). Immediately after transplantation, islets are exposed to hypoxia due to delayed revascularization, leading to a decreased supply of nutrients to *β* cells at the islet core. However, we did not observe protection of islets when autophagy was induced by trehalose, rapamycin and salt solution during hypoxia (Figure 3(b)). Next, we attempted an autophagic preconditioning experiment with the inclusion of other new inducers. There was indeed significant protection in these cells when exposed to hypoxia as shown by decrease in the activation of caspase-3 (Figure 3(c)). Especially, FDA-approved drugs including niguldipine and penitrem A showed significant protection. Islets immediately following isolation are likely to display defects in the autophagic machinery and are likely to succumb to hypoxia-induced cell death after transplantation. Thus, there is a need to restore the normal autophagic pathway in cultured islets before transplantation. Current understanding in the islet transplantation field is that it is desirable to culture islets for 24–72 h before transplantation since islets can recover from isolation-induced stress and immunogenicity of the islets is reduced. This culture period could also be used to assess and manipulate autophagic pathway to improve transplantation outcome.

The pancreatic *β* cells are particularly vulnerable to oxidative stress-induced injury due to low-level expression of antioxidant enzymes [32, 33]. Markers of oxidative stress and cellular fragility (RBC) have been shown to be elevated in nondiabetic relatives of type 1 diabetic patients [34]. It has been reported that *β* cell apoptosis could be significantly reduced in NOD mice, an autoimmune diabetic animal model, after administration of an MnSOD mimetic that is known to scavenge free radicals [35]. Proinflammatory cytokines induce the expression of iNOS through NF-*κ*B and GAS sites in iNOS promoter leading to the generation of nitric oxide [36]. When nitric oxide combines with superoxide generated by macrophages or cytokines, highly toxic peroxynitrite is generated. Several studies have demonstrated that peroxynitrite is an important mediator of cytokine-induced *β* cell death in type 1 diabetes [37–39]. In addition, ceramide is known to be one of the mediators of cytokine action. The decrease of active form of Akt by ceramides can interfere with growth factor action on CREB. We observed that peroxynitrite and C2, a synthetic ceramide, mimic cytokine-induced apoptosis pathway (Table 1 and Figure 5).

Although CREB elicits antiapoptotic pathways in *β* cells, its function can be impaired by other transcription factors in diabetes. For example, JNK and its nuclear target, c-jun, have been shown to be mediators of *β* cell-death. JNK-activated c-jun induces the expression of stress-associated genes [40]. Cytokines strongly stimulate the signaling pathways leading to JNK activation [26]. Cell-permeable peptide inhibitors of JNK block cytokine-induced *β* cell death [41]. Inhibition of JNK is being considered as an important strategy to improve *β* cell survival. We have reported that SP600125, a JNK inhibitor, activates CREB [42].

ER stress is known to interact with the pathways of apoptosis and autophagy. For example, Islet amyloid polypeptide (IAPP) which is colocalized and secreted with insulin from *β* cell granules [43] induces *β* cell apoptosis in cultured islets and in type 2 diabetes [44–46]. ER stress is the main mechanism through which IAPP aggregates cause *β* cell apoptosis [47, 48]. Silencing of IAPP with siRNA improves the survival of cultured human islets [49]. ER stress is also a potent inducer of autophagy [50, 51]. It has been suggested that autophagy might represent a protective cellular response under conditions of ER stress [52]. We observed formation of LC3-II, a marker for autophagy in MIN6 cells following induction of ER stress with thapsigargin and tunicamycin (Figure 2(c)). ER stress inducers also activated caspase-3 which was significantly reduced by exendin-4, a GLP-1 analogue (Figure 4(c)).

It this study, we demonstrate that multiple stresses, generated during islet isolation and transplantation, induce *β* cell death by orchestration of gene expression patterns in apoptosis pathway. Although a parallel cell survival pathway is also activated, it is overwhelmed by stress-induced proapoptotic genes. Autophagy, a physiological cytoprotective pathway, interacts with apoptosis when induced in excess in the islet transplantation setting. Therefore, multiple approaches are needed to improve islet transplantation outcome.

## Figures and Tables

**Table 1:** Functional grouping of apoptosis pathway genes.

Bcl-2 family	BAD, BAG1, BAG3, BAG4, BAK1, BAX, BCL2, BCL2A1, BCL2L1, BCL2L10, BCL2L11, BCL2L2, BCLAF1, BID, BIK, BNIP1, BNIP2, BNIP3, BNIP3L, HRK, MCL1
Caspase family	CASP1, CASP10, CASP14, CASP2, CASP3, CASP4, CASP5, CASP6, CASP7, CASP8, CASP9
IAP family	BIRC1, BIRC2, BIRC3, BIRC4, BIRC6, BIRC8
TRAF family	TRAF2, TRAF3, TRAF4
CARD family	APAF1, BCL10, BIRC2, BIRC3, CARD4, CARD6, CARD8, CASP1, CASP2, CASP4, CASP5, CASP9, CRADD, NOL3, PYCARD, RIPK2
Death domain family	CRADD, DAPK1, FADD, FAS (TNFRSF6), TNFRSF10A, TNFRSF10B, TNFRSF11B, TNFRSF1A, TNFRSF21, TNFRSF25, TRADD
Death effector domain family	CASP8, CASP10, CFLAR, FADD
CIDE domain family	CIDE-A, CIDE-B, DFFA
p53 and DNA damage response	ABL1, AKT1, APAF1, BAD, BAX, BCL2, BCL2L1, BID, CASP3, CASP6, CASP7, CASP9, GADD45A, TP53, TP53BP2, TP73
Anti-apoptosis	AKT1, BAG1, BAG3, BCL2, BCL2A1, BCL2L1, BCL2L10, BCL2L2, BFAR, BIRC1, BIRC2, BIRC3, BIRC4, BIRC6, BIRC8, BNIP1, BNIP2, BNIP3, BRAF, CFLAR, IGF1R, MCL1, TNFRSF7
TNF ligand family	CD40LG (TNFSF5), FASLG (TNFSF6), LTA, TNF, TNFSF10, TNFSF7, TNFSF8
TNF receptor family	CD40 (TNFRSF5), FAS (TNFRSF6), LTBR, TNFRSF10A, TNFRSF10B, TNFRSF11B, TNFRSF1A, TNFRSF1B, TNFRSF21, TNFRSF25, TNFRSF7, TNFRSF9

**Table 2:** Mean fold (>2.0) changes in apoptosis pathway gene expression in islets after exposure to oxidative stress (H_2_O_2_ and peroxynitrite), hypoxia, and cytokines.

Genes	Role in apoptosis	Peroxynitrite	H_2_O_2_	Hypoxia	Cytokines
Bcl2A1	Anti-apoptotic; Bcl2 family	3.2	2.1	2.4	11.4
Bid	Proapoptotic; BH3 only	3.7	3.5	2.5	3.4
Fas	Extrinsic; receptor	4.3	3.1	3.4	7.4
Fas ligand	Extrinsic; receptor ligand	2.4	2.1	NS	3.4
TRAIL	Extrinsic; receptor ligand	4.6	3.6	NS	4.2
A20	Extrinsic; anti-apoptotic	2.3	NS	2.5	7.4
c-Flip	Extrinsic; anti-apoptotic	2.6	NS	NS	4.2
Caspase-3	Marker for apoptosis	4.5	3.2	3.2	2.3
BIRC3	Caspase inhibitor	3.2	2.4	NS	8.7
CARD8	Caspase-9 inhibitor	−2.3	−2.4	−2.1	−2.3
BRAF	Signaling kinase	−3.7	−3.5	−2.5	NS
BFAR	Anti-apoptotic; links both pathways	−4.8	−3.5	−4.2	NS
CIDE-A	Causes DNA fragmentation	3.2	2.4	3.8	NS
BNIP3	Autophagy Inducer	2.6	NS	5.6	NS

Human islets (2000 IEQ) were exposed to 200 *μ*M of peroxynitrite, 200 *μ*M of H_2_O_2_ or a mixture of cytokines (2 ng/mL of IL-1*β*, 10 ng/mL of TNF-*α* and 10 ng/mL of IFN-*γ*) for 24 h or cultured under hypoxic conditions (1% oxygen) for 8 h. The cDNA synthesized from isolated RNA was mixed with Master Mix containing SYBR Green and distributed into 96 wells containing primers for the 84 genes associated with the apoptotic pathway. Five housekeeping control genes and three RNA and PCR quality controls were also included. PCR analysis was carried out and the fold changes between control and treated were calculated based on Δ*C*_*t*_ and corrected for GAPDH expression. Results are the mean obtained from four different batches of human islets. NS: not significant.
